# Seasonal dynamics of *Rhipicephalus rossicus* attacking domestic dogs from the steppic region of southeastern Romania

**DOI:** 10.1186/1756-3305-7-97

**Published:** 2014-03-10

**Authors:** Mirabela Oana Dumitrache, Botond Kiss, Filipe Dantas-Torres, Maria Stefania Latrofa, Gianluca D’Amico, Attila David Sándor, Andrei Daniel Mihalca

**Affiliations:** 1 Department of Parasitology and Parasitic Diseases, University of Agricultural Sciences and Veterinary Medicine Cluj-Napoca, Calea Mănăştur 3-5, Cluj-Napoca 400372, Romania; 2 Danube Delta National Institute for Research and Development, Strada Babadag 165, Tulcea 820112, Romania; 3 Department of Immunology, Aggeu Magalhães Research Centre, Oswaldo Cruz Foundation 50670420, Recife, Pernambuco, Brazil; 4 Department of Veterinary Medicine, University of Bari, 70010 Valenzano, Bari, Italy

**Keywords:** *Rhipicephalus rossicus*, Dogs, Danube delta biosphere reserve

## Abstract

**Background:**

Danube Delta Biosphere Reserve is one of the most interesting regions in Europe from an epidemiological point of view due to its great biodiversity, local climatic conditions and various types of habitats. Moreover, there is no data regarding the ectoparasite communities of dogs from this area. In this frame, the aims of our study were to establish the tick communities parasitizing dogs and to provide new data regarding seasonal dynamics of a neglected tick species, *Rhipicephalus rossicus.*

**Methods:**

A survey was carried out in order to gather information regarding tick species attaching to domestic dogs from a steppic region of southeastern Romania and to establish their seasonal dynamics. The research was conducted from 1 December 2012 to 30 November 2013, on 8 dogs from Iazurile, a locality from the west-central part of the Danube Delta Biosphere Reserve. In total, 384 examinations were made, each dog being checked for tick infestation 4 times per month, for one year.

**Results:**

The 893 ticks found belonged to six species: *R. rossicus* (95.6%), *Dermacentor reticulatus* (3.2%), *Ixodes ricinus* (0.5%), *Hyalomma marginatum* (0.3%), *Rhipicephalus sanguineus* sensu lato (s.l.) (0.2%) and *Ixodes crenulatus* (0.1%). From the 91 positive examinations, *R. rossicus* was found in 80 (87.9%). Single species infestation occurred in 84 examinations. In 7 out of 91 positive examinations mixed infestation were found. No ticks were found in December, January and September.

**Conclusions:**

For *R. rossicus*, high frequency and intensity were observed in May, June and July. The activity peaks for *D. reticulatus* were in spring and autumn. Our results highlight that within the range of *R. sanguineus* s.l., the most common dog tick worldwide, selected dog populations may be predominantly infested by closely related species, like in our case, *R. rossicus*.

## Background

Ticks, important vectors for human and animal pathogens, have shown an increased spread across the world [1], probably as a consequence of the enhanced mobility of domestic animals, of the ability of ticks to find niches in new climatic conditions and due to the growing accessibility of natural environments [2]. The awareness on these arthropods is highlighted by the rapid advancement in molecular techniques that enables detection of vector-borne pathogens [3]. The increasingly close relationship between dogs and humans, correlated with the fact that some canine tick-borne pathogens are causing zoonotic diseases, poses new concerns for veterinary and human public health and draws both clinical and scientific attention on ticks.

Temporospatial distribution of ticks is closely related to the risk for the transmission of various pathogens [4]. Monitoring the tick populations in a given area is one of the fundamental steps in the assessment of transmission risks of tick-borne pathogens. The most exhaustive review on the distribution of hard-ticks in Romania was published almost 50 years ago [5]. In the few last years several studies [6-9] have been made but there is still poor information about hard tick species occurring on dogs and even scantier information regarding tick species seasonality. The tick fauna of Romania consists of 25 species [8]. However, detailed information on their distribution, host preferences and seasonal dynamics is available only for a few. Six species of hard ticks have been reported so far on dogs in Romania: *Ixodes ricinus*, *Haemaphysalis punctata*, *H. concinna*, *Dermacentor marginatus*, *Rhipicephalus sanguineus* sensu lato (s.l.) and *Hyalomma marginatum*[8]. Some of these ticks have a more general geographical distribution (e.g. *I. ricinus*, *D. marginatus*, *H. punctata*) while others are limited to the warmer part of the country (*H. concinna*, *R. sanguineus* s.l., *H. marginatum*). Moreover, this spatial distribution pattern was shown to influence the epidemiology of certain tick-borne pathogens of dogs [10].

One of the most interesting regions from Europe from an epidemiological point of view is the Danube Delta Biosphere Reserve. Its importance resides in the great biodiversity, diversity of habitats and rural lifestyle of local people, with limited veterinary services available, mainly for dogs [11]. Moreover, the ectoparasite communities of dogs were never studied in detail here, mainly in the steppe ecoregions. In the frame of climate change, globalization, zoogeographical range extension for both hosts and ticks, and an increase in pet mobility, studies regarding spatial and temporal occurrence of ticks are particularly important. The aims of this study were to establish the tick communities parasitizing dogs and to provide new data regarding seasonal dynamics of a neglected, but widely distributed steppe tick, *Rhipicephalus rossicus* in a locality from the steppic region of Danube Delta Biosphere Reserve.

## Methods

### Study area

**Figure 1:**
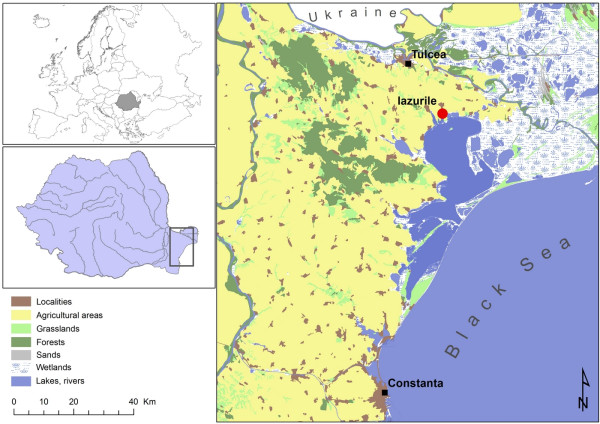
Study site: geographic positioning of the locality Iazurile.

The study site, Iazurile (45.014040 N, 28.941084 E) (Figure 1) from Tulcea County, lays in the west-central part of the Danube Delta Biosphere Reserve, a wetland complex situated in SE Romania, close to the western coast of the Black Sea. The region is in the western corner of the steppe bioregion and it is a mixture of wetlands and steppic grasslands (mostly converted into arable lands). There are a number of freshwater and brackish wetlands dominated by reed (*Phragmites australis*) with extensive agricultural lands and a number of small villages in between [11]. The Carpathian Mountains serve as a barrier and block the continental influences of the vast plains from the north, which results in frosty winters and less rain to the south and southeast of the country. In the extreme southeast, the influence of The Black Sea offers a milder, maritime climate [12]. The climate is continental with a mean temperature in January of -1.8°C and 22.2°C in August. Most lakes and slow flowing branches/channels freeze in winter, but ice cover lasts for short periods only. The precipitation is mostly in the form of rain in spring and autumn, with a yearly average of 350 mm and the evaporation is about 1000 mm/year. The local human population is involved in fishery and subsistence agriculture for living, with small herds of livestock (mainly sheep which use the nearby pastures) being common at family level. Most households have one or more dogs [11].

### Study design, tick collection and identification

A total of 8 dogs, (2 Hungarian Vizsla and 6 mixed breed), privately owned by locals from Iazurile village, were observed from 1 December 2012 to 30 November 2013. All dogs had a high degree of roaming. The dogs or the environment have never been treated before or during the sample collection with products active against ticks.

All dogs were examined by gross inspection of the entire skin surface four times per month during the whole period of the study. All ticks were collected from each dog, regardless of species and life stage, stored in separate tubes containing pure ethanol and brought to the laboratory of the Department of Parasitology and Parasitic Diseases, USAMV Cluj-Napoca. All ticks were counted, separated by developmental stage and gender (adults) and identified to species level by using morphological keys [5,13-16] and descriptions [1,17] under a binocular microscope. Moreover, two representative specimens of *R. rossicus* (the most abundant species found; see *Results*) were also molecularly identified.

Briefly, the DNA extraction was performed using a commercial kit (DNeasy Blood & Tissue Kit, Qiagen GmbH, Hilden, Germany), in accordance with the manufacturer’s instructions. Partial gene sequences of mitochondrial 12S rDNA (∼400 bp) were generated and analyzed. Primers and PCR conditions have been described elsewhere [1]. The amplicons were purified and sequenced directly using the Taq DyeDoxyTerminator Cycle Sequencing Kit (v.2, Applied Biosystems) in an automated sequencer (ABI-PRISM 377). The nucleotide sequences obtained were aligned and edited using BioEdit software Version 7.1.3.0 [18] and compared among them and with those available in GenBank dataset by Basic Local Alignment Search Tool (BLAST –http://blast.ncbi.nlm.nih.gov/Blast.cgi).

### Statistical analysis

Mean intensity and frequency were calculated using the EpiInfo 2000 software.

## Results

After a one-year surveillance and 48 examinations of each one of the 8 dogs (in total 384) in the study, 91 examinations yielded ticks with 893 specimens collected. Intensity of infestation ranged from one to 60 ticks per dog per examination. The majority (892; 99.9%) of the specimens were adults belonging to six species. *Rhipicephalus rossicus* was the most common (95.6%, n = 854), followed by *Dermacentor reticulatus* (3.2%, n = 29), *I. ricinus* (0.5%, n = 4), *H. marginatum* (0.3%, n = 3), *R. sanguineus* s.l. (0.2%, n = 3) and *I. crenulatus* (0.1%, n = 1). Only one nymph (*I. ricinus*) (0.01%) was identified.

From the 91 positive examinations, *R. rossicus* was found in 80 (87.9%), being the most frequent tick species, followed by *D. reticulatus* which was present in 12 examinations (13.2%). *Ixodes ricinus* and *R. sanguineus* s.l. occurred in 4 (4.4%) and in 2 (2.2%) of the positive checks, respectively, whereas *H. marginatum* and *I. crenulatus* in only one (1.1%). Single species infestation occurred in 84 examinations. There were 5 mixed infestations with 2 tick species. The most frequent (n = 3) association was between *R. rossicus* and *D. reticulatus*. The other two cases of co-infestation were *D. reticulatus* + *I. ricinus* and *R. rossicus* + *R. sanguineus* s.l., each one represented by one case. Mixed infestations with 3 tick species were detected in two cases: *D. reticulatus* + *R. rossicus* + *I. ricinus* and *R. rossicus* + *R. sanguineus* s.l. + *H. marginatum*.

**Figure 2:**
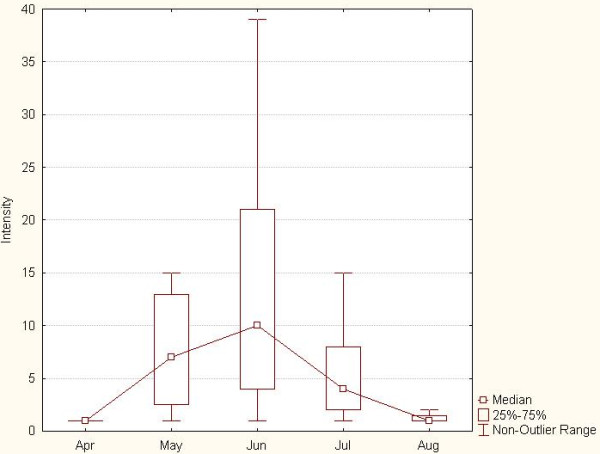
**The mean intensity of *R. rossicus *** **parasitism in the studied period (mean, ±95% confidential intervals).**

The monthly distribution of the collected tick species is shown in Table 1. The distribution of *R. rossicus* is highly grouped, with first ticks collected in late April, and the last ones observed in August. High frequency was observed in May, June and July, peaking in late June. The intensity also peaked in June (mean intensity 10.1, SD = 13.2), and decreased abruptly until August (Figure 2).

**Table 1:** Number of specimens from the indicated species according to month of collection (- = zero)

Month*	**Total**	***R. rossicus***	***R. sanguineus *** **s.l.**	***I. ricinus***	***I. crenulatus***	***D. reticulatus***	***H. marginatum***
**February**	1	-	-	-	-	1	-
**March**	12	-	-	2	-	10	-
**April**	14	4	-	1	1	8	-
**May**	206	199	1	-	-	3	3
**June**	513	510	1	1	-	1	-
**July**	131	131	-	-	-	-	-
**August**	10	10	-	-	-	-	-
**October**	5	-	-	-	-	5	-
**November**	1	-	-	-	-	1	-
	893	854	2	4	1	29	3

*No ticks were found in December, January and September.

BLAST analysis of 12S rDNA sequences of ticks morphologically identified as *R. rossicus* showed a 99% nucleotide identity with a reference sequence of *R. rossicus* (AF150021) available in GenBank. The 12S rDNA nucleotide sequence of *R. rossicus* generated in this study has been deposited in GenBank database (Accession number KJ425484).

## Discussion

Each geographical region has its own climatic characteristics and habitat types that will make it preferable to certain species of animals and ticks and consequently will define the tick-borne pathogen spectrum for that particular area. The encroachment and the population increase of certain wild species coupled with urbanization together with the ability of ticks to extend their distribution range highlights the importance of permanent research on ticks and tick-borne diseases. In those areas where the last comprehensive studies were made decades ago this need becomes a must.

Unpredictably, *R. rossicus* was the predominant tick species of dogs in our study. Pomerantzev *et al*. [19] consider it a typical representative of the steppe and mountain-steppe areas in a narrow sense and define its distribution zone in Ukraine, lower Volga region, northern Kazakhstan up to Semiretchie, Ciscaucasia, Transcaucasia, Turkmenistan and border districts between Armenia and Turkey. Kolonin [20] redraws the distribution range of *R. rossicus*: Bulgaria, Romania, Moldavia, Ukraine, Russia (Rostov, Voronezh, Saratov, Volgograd, Astrakhan and Orenburg Oblasts, Krasnodar and Stavropol Krays, Republic: North Ossetia, Kalmykia, Chechen, and Dagestan), Georgia (eastern), Armenia, Azerbaijan, Kazakhstan, Turkmenistan, Uzbekistan, Iran, China (Xingjiang), and Egypt (Sinai). Regarding the presence of this tick species in Romania, Feider [5] states that *R. rossicus* can be found only in Dobrogea (SE of the country). Since then, there is only one report of this species in Romania, from *Erinaceus roumanicus*[8].

Detailed information regarding the host spectrum and seasonality of *R. rossicus* in Ukraine is provided by Emchuk [21]. According to these data, in Ukraine *R. rossicus* was found in 25 hosts. The larval parasitism was recorded on 15 host species from March to November. Nymphs were found on 22 host species from March to November while adults occurred all year around. In the case of dogs, adult ticks were found from March to November and nymphs only in July and August. According to Pomerantzev *et al*. [19] in Transcaucasia adults of *R. rossicus* are active from April to September. Gusev *et al*. [22] collected adults from birds from Kura-Araksinsky lowlands, Azerbaijan in April, June and November. Several authors have reported the maximum peak of activity in June and July [23]. The present study reveals consistent aspects regarding *R. rossicus* seasonal activity and confirms that in Romania this tick maintains the seasonal patterns found in other regions. Although in our study *R. rossicus* was the most common dog tick, there are surprisingly few reports on dogs worldwide [15,24]. This might be explained by various situations: lack of extensive studies on dogs from Eurasian steppe regions, preferential studies during non-activity seasons or misidentification with closely related species (i.e., *R. sanguineus* s.l.).

*Rhipicephalus rossicus* is a vector of the Crimean-Congo hemorrhagic fever virus, *Francisella tularensis* and *Coxiella burnetii*[15]. In the light of the first serologic evidence for the circulation of Crimean-Congo hemorrhagic fever virus in Romania, in Tulcea County [25], our research highlights the importance of the common presence of another potential vector for CCHFV, *R. rossicus.* This data is of eco-epidemiological significance an will contribute to the development of prediction maps. Establishing and understanding disease epidemiology helps us to prevent the spread and transmission of pathogens which are dangerous to both animals and humans. According to Akimov & Nebogatin [24], we are the witnesses of the invasion of *R. rossicus* into the areas previously not inherent to it. *Canis familiaris* is a new host recorded for *R. rossicus* in Romania. Incidentally, *R. rossicus* was originally described based on specimens collected from hedgehogs and rats in the governmental district of Saratov, Russia [26]. Altogether, available data suggests that dogs might be important hosts for adult *R. rossicus* in some areas where this tick occurs. Nonetheless, further research is needed to understand the actual distribution and host range of *R. rossicus*.

*Dermacentor reticulatus*, a well-known vector of *Babesia canis*, tick-borne encephalitis virus, *Francisella tularensis* and *Rickettsia* spp. [27,28] is widely distributed throughout the temperate zones of Eurasia. Its distribution is not continuous and is divided into two parts, western and eastern European. The eastern European part begins in eastern Poland and Slovakia, and extends through Ukraine, eastern Hungary and Romania, Belarus and Russia to Siberia. It seems that the distribution range of *D. reticulatus* expands to higher latitudes and altitudes [29]. In Romania there are some reports from the northern part of the country [5] and recently it was reported in western Romania [30]. This study is the first report of *D. reticulatus* from southeastern Romania. Our findings bring new evidence for the expanding distribution of *D. reticulatus* and explain the increasing prevalence of *B. canis* in our country in the last few years, especially in dogs from the southern region [31].

Interestingly, *R. sanguineus* s.l., accounted as the most widespread tick of dogs and a well-recognized vector of numerous pathogens of dogs and humans [1] was poorly represented; only two specimens were found. We can conclude that *R. rossicus* and not *R. sanguineus* s.l. is the dominant tick species of dogs from steppic regions of southeastern Romania.

Although *I. ricinus* is widespread in Europe [32] and also in Romania [33] and has a wide host spectrum, in this study it was found in a reduced number, probably due to its habitat preference. The few specimens of *H. marginatum* and the single one of *I. crenulatus* indicate that these species are found on dogs only accidentally.

## Conclusions

The present paper highlights the importance of detailed specific diagnosis of ticks collected from dogs, considering that, within the range of *R. sanguineus* s.l., certain dog populations may be predominately infested by closely related species, like in our case *R. rossicus*. As the vectorial role of *R. rossicus* for important canine pathogens has not been evaluated yet, our results suggest more detailed investigations on this topic.

## Competing interests

The authors declare that they have no competing interests.

## Authors’ contributions

MOD wrote the manuscript and identified the ticks. BK collected samples. FDT and MSL performed the molecular identification of tick species. GD collected samples and identified ticks. ADS made the statistical analysis and sample collection. ADM coordinated the research team. All authors read and approved the final manuscript.
